# Comment on the CIBCD model for chronic critical illness

**DOI:** 10.1186/s13054-025-05471-y

**Published:** 2025-07-08

**Authors:** Valery V. Likhvantsev, Mikhail Ya. Yadgarov, Alexey A. Yakovlev, Levan B. Berikashvili

**Affiliations:** 1 Federal Research and Clinical Center of Intensive Care Medicine and Rehabilitology, Moscow, Russia; 2 First Moscow State Medical University, Moscow, Russia

We read with great interest the recent article by Mechanick et al. [1], which introduces the Critical Illness-Based Chronic Disease (CIBCD) model as a conceptual framework for chronic critical illness (CCI). We were pleased to see that the authors referenced our Tri-Steps Model [2] as part of the broader discussion on CCI evolution. While we welcome this contribution, we wish to raise several concerns regarding the structure of the proposed model, specifically its inclusion of the initial “risk” stage and the final “complication” stage. We suggest that these stages may blur rather than clarify clinical decision-making. First, the “risk” stage encompasses virtually all critically ill patients, who inherently possess risk factors for CCI. As such, it does not represent a meaningful clinical inflection point. Second, the “complication” stage appears to describe inevitable consequences of disease trajectory rather than a distinct, actionable phase. These risks diluting the model's utility for bedside clinicians. By contrast, our Tri-Steps Model delineates three discrete and clinically observable phases of CCI progression based on physiologic transition points—principally inflammation, catabolism, and immunosuppression. We believe this approach provides a more pragmatic structure for clinical application and patient stratification. We commend the authors for advancing the conversation around CCI phenotyping and invite further refinement through integrative dialogue among researchers and clinicians.

## Data Availability

Not applicable as there are no data. The comments concern a paper that is accessible on PubMed.

## Abbreviations

CCI: Chronic critical illness
CIBCD: Critical illness-based chronic disease
